# The propeptide of human thyroid peroxidase is not required for its cellular, enzymatic or immunological activity

**DOI:** 10.1186/1756-6614-6-S2-A19

**Published:** 2013-04-05

**Authors:** Marlena Godlewska, M Góra, AM Buckle, BT Porebski, EH Kemp, W Krasuska, BJ Sutton, JP Banga, Barbara Czarnocka

**Affiliations:** 1 Medical Centre of Postgraduate Education, Department of Biochemistry and Molecular Biology, Warsaw, Poland; 2 Institute of Biochemistry and Biophysics PAS, Department of Genetics, Warsaw, Poland; 3 Department of Biochemistry and Molecular Biology, Faculty of Medicine, Monash University, Clayton, Australia; 4 Department of Human Metabolism, School of Medicine Sheffield, University of Sheffield, UK; 5 Randall Division of Cell & Molecular Biophysics, King’s College London, London, UK; 6 Division of Diabetes and Nutritional Sciences, King's College London, London, UK

## 

Thyroid Peroxidase (TPO), a dimeric, membrane-bound glycoprotein found in the thyroid follicular lumen, catalyses the biosynthesis of thyroxine, and is also a major autoantigen in autoimmune thyroid disease (AITD). TPO molecules undergo posttranslational modifications such as glycosylation, heme incorporation, dimer formation, and proteolytic processing in the N-terminal region. Due to propeptide deletion, in human thyroid cells the TPO protein loses 108 amino acids in the N-terminus to yield mature enzyme. During the course of studies to obtain homogenous preparations of recombinant TPO for structural studies, we investigated the role of the propeptide in TPO structure, function and trafficking. We engineered recombinant human TPO truncated at the N-terminal residue (TPO-pro) and subsequently expressed in Chinese hamster ovary (CHO) cells. Then we characterized TPO-pro and compared its properties with wild-type recombinant TPO (TPOwt) based upon its subcellular localisation, immunological and biochemical properties such as glycosylation and enzymatic activity. Molecular modeling and dynamics simulation of TPO-pro comprising a dimer of MPO-like domains were performed in order to investigate the impact of propeptide removal. Using immunodetection and densitometric analysis we showed that deletion of the propeptide did not influence expression level of TPO-pro in comparison with TPOwt. Upon SDS-PAGE under non-reducing conditions, we observed dimer formation for both TPOwt and TPO-pro. Furthermore, we tested by flow cytometry the reactivity of TPO constructs with anti-TPO antibodies and confirmed that the propeptide deletion did not disturb TPO transport to the membrane of CHO cells. This finding was further supported by immunocytochemistry. ELISA and flow cytometry analysis revealed that the conformation of epitopes recognized by a panel of human anti-TPO antibodies was not considerably affected in TPO-pro. Measurement of the Amplex Ultra Red oxidation of cells expressing TPOwt and TPO-pro confirmed faithful enzymatic activity of extracellular truncated TPO. Study of the carbohydrate content showed that TPO-pro is effectively N-glycosylated. Molecular modelling and dynamics simulations were consistent with our findings. In conclusion, the propeptide is not essential for the proper folding and transport of TPO produced in CHO cells. The successful expression in a membrane-anchored, active form that is insensitive to intramolecular proteolysis is a key advance for purification of substantial quantities of homogenous preparation of TPO for structural and crystallization studies.

